# Mystery mushroom malingerers: *Megaselia marquezi* Hartop et al. 2015 (Diptera: Phoridae)

**DOI:** 10.3897/BDJ.5.e15052

**Published:** 2017-08-28

**Authors:** Brian V. Brown, Emily A. Hartop

**Affiliations:** 1 Natural History Museum of Los Angeles County, Los Angeles, United States of America

**Keywords:** Natural history, Diptera, fungivore, urban, biodiversity, citizen science

## Abstract

A mysterious female phorid fly, known for many years to be associated with fungal sporophores ("mushrooms") is identified as *Megaselia marquezi* Hartop et al. 2015. Male and female flies were collected emerging from the fungus *Psathyrella candolleana* (Fr.) Maire, and females were observed swarming over the sporophores.

## Introduction

The genus *Megaselia* Rondani 1856 (Diptera: Phoridae) is immense, with over 1000 described species and certainly thousands more unrecognized. In addition to their species-richness, the lifestyles of various species are so diverse and little-studied that any addition to this knowledge is of great significance.

For nearly thirty years, one of us (BVB) has been aware of an unusual species of *Megaselia* whose females were associated with fungal fruiting bodies. These females have an unusual ovipositor that would lead them to key unconvincingly to *Plastophora* Brues 1905 (now mostly subsumed in *Myriophora* Brown 1993) in the key of Borgmeier (1963). Males were unknown, and had not been found on the fungi. The distinctive females (or ones similar to them) were found in eastern Canada, Costa Rica, and Los Angeles, California, USA.

Meanwhile, a large inventory project in Los Angeles has led to an unprecedented knowledge of the urban phorids of this city (Brown and Hartop 2016, Hartop et al. 2015, Hartop et al. 2016a, Hartop et al. 2016b). About 100 species, mostly of *Megaselia*, are known from Los Angeles, but many were new to science and had nothing known of their lifestyle. Matching a lifestyle with a species previously known only from a name is a significant accomplishment.

## Material and methods

Specimens were collected from the back garden of a commercial bed and breakfast establishment in downtown Los Angeles, near the campus of the University of Southern California (coordinates 34.02 degrees North, 118.17 degrees West). On 20 April 2017, large numbers of fruiting bodies of the fungus *Psathyrella candolleana* (Fr.) Maire were noted in the garden. Adult flies that were flying above or running on the fungi (as in Fig. 1) were collected into 70% ethanol. Specimens were collected from two locations in the garden, one of which yielded teneral specimens that were apparently just emerging from the soil surrounding the fungi.

Several fruiting bodies of the fungi were removed and brought in to the laboratory for further study.

The BioSCAN project (Brown and Hartop 2016) is an ongoing survey of insects in urban Los Angeles, as part of the investigations of the Natural History Museum of Los Angeles County's Urban Nature Research Center. Data from phase I of the BioSCAN project were reviewed for placing the current finding about *Megaselia marquezi* Hartop et al. (2015) in context. Correlation analysis was done using Microsoft EXCEL on the entire data set for the BioSCAN project.

## Results

**Figure 2.**
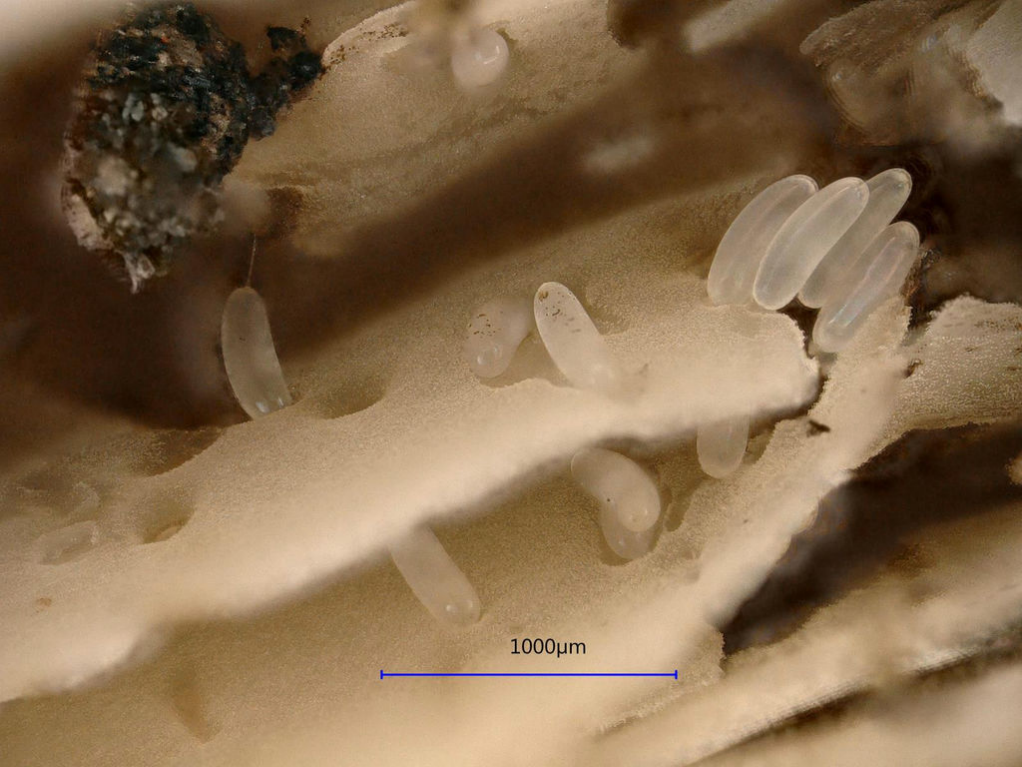
*Megaselia marquezi* Hartop et al. eggs laid in the gills of the *Psathyrella candolleana* (Fr.) Maire.

Flies on fungi were noted in early April, 2017. Specimens of flies were collected 20 April, and fungi were brought to our laboratory on 24 April, 2017. On fully open fungal sporophores (“mushrooms”), we noted large numbers of eggs laid within the gills (Fig. 2). Two adult female flies were observed feeding on the mushrooms (Fig. 3, Fig. 4). One of the females was exposed when we broke open the cap of a mushroom that was still closed, it appeared from her behavior that she may have just oviposited (Fig. 5). Larvae were found feeding on the lower surface of the mushroom cap, deep within the gills (Fig. 6). One large mushroom (ca. 5 cm in radius) was estimated by subsampling to contain at least 500 larvae. One puparium was found in surrounding soil we collected onsite. The soil and mushrooms were kept for several weeks at room temperature in a plasltic bag, and larvae were found to have exited the mushrooms to pupate in the soil within a few days.

We observed hundreds of flies around and on the fungi, on nearby leaf litter and on soil. Among the teneral flies emerging from the soil surrounding the fungi were many females and three males of the “mystery” phorid, identified (from the males) as *Megaselia marquezi* Hartop et al. Because only females are attracted to the fungi, the collection of teneral specimens, including males, was vital to this discovery.

Most of the specimens of this fly collected by the survey Malaise traps (1715 in total) were caught at a single site in April, 2015, when a sample contained 459 individual males (459/1715 = 27%) .

## Discussion

**Figure 7.**
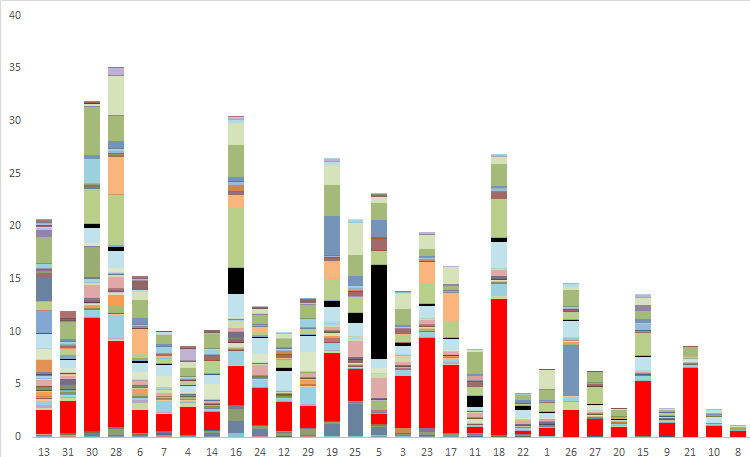
Stacked bar chart of phorid fly catch from one year of sampling at each of 30 sites from BioSCAN project. Each color represents a different species, each bar represents a single site. Black - *Megaselia marquezi* Hartop et al.; red - *Megaselia agarici* (Lintner). Vertical axis- number of speciemens; horizontal axis- trapping sites (see Brown and Hartop 2016).

Among over 42,000 phorid flies collected by BioSCAN project in Los Angeles (Hartop et al. 2015) , *Megaselia marquezi* was the sixth most commonly collected species, occuring in all but two sites in our study. Its occurrence is most highly correlated to that of *M. berndseni* (Schmitz, 1919), a species also known to breed in the fungus *Psathyrella candolleana* in Europe (Table 1). Other phorids known to breed in this fungus are noted in Table 1. The most frequently collected fly in the survey, *M. agarici* (Lintner, 1895) (Fig. 7), is also a fungivore.

**Figure 8.**
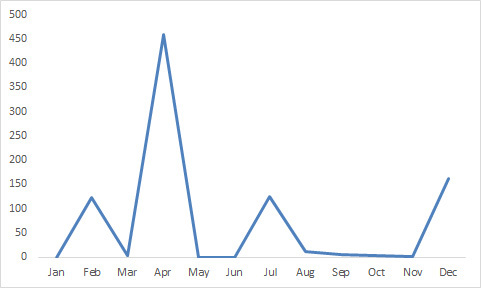
Numbers of specimens of male *Megaselia marquezi* Hartop et al. at BioSCAN site 5 throughout 2015.

Although *Megaselia marquezi* was recorded in many of the BioSCAN trap samples, its ranking as the sixth most common species in the survey is largely based on the huge outburst of specimens found in trap number 5 in April 2015 (Figs 7, 8). Based on our observations of the masses of adults and larvae at the site reported herein, however, this surge of individuals could have been caused by a small group of mushrooms emerging near the trap. Caution is needed when interpreting numbers of phorid flies in trap samples when such scattered resources as mushrooms can lead to spikes in numbers of specimens in the BioSCAN traps.

## Conclusions

So far, in spite of our incomplete knowledge of *Megaselia* life histories, we can say that the urban fauna of Los Angeles is numerically dominated by fungus-feeders (Brown and Hartop 2016), including the newly discovered lifestyle of *M. marquezi*. Of the 42,480 specimens identified, 18,918 (45%) are now known to be of species that are fungivores. Possibly, the widespread irrigation of lawns allows fungal growth that supports an abundant fungivore community, but our ignorance of the fauna of the surrounding natural areas makes such statements highly speculative.

## Figures and Tables

**Figure 1.** *Megaselia marquezi* Hartop et al. hovering over *Psathyrella candolleana* (Fr.) Maire.

**Figure 3.** A female *Megaselia marquezi* Hartop et al. feeding on *Psathyrella candolleana* (Fr.) Maire. A single larva can be seen feeding in the tissue beneath her.

**Figure 4.** A close-up of a female *Megaselia marquezi* Hartop et al. feeding on *Psathyrella candolleana* (Fr.) Maire.

**Figure 5.** *Megaselia marquezi* Hartop et al. inside the broken-open cap of a closed *Psathyrella candolleana* (Fr.) Maire. Based on the extended abdomen, she appears to have just oviposited.

**Figure 6.** *Megaselia marquezi* Hartop et al. larvae feeding in the gills of the *Psathyrella candolleana* (Fr.) Maire.

**Table 1.** *Megaselia* species known to be associated with the fungus *Psathyrella candolleana*.

*Megaselia* species	Region or country	Reference
*M. berndseni* (Schmitz, 1919)	England	Disney and Evans 1979, Disney and Evans 1990
*M. hirtiventris* (Wood, 1909)	England	Disney and Evans 1999
*M. latior* Schmitz.1936	England	Disney and Evans 1999
*M. nigra* (Meigen 1830)	England	Disney and Evans 1999
*M. okazakii* Disney, 2014	Japan	Disney 1989
*M. rufipes* (Meigen, 1804)	Europe	Eisfelder 1956
*M. spinicincta* (Wood, 1910)	Europe	Eisfelder 1956
